# Supplementary figures and images for: Dynamic genetic architecture of yeast response to environmental perturbation shed light on origin of cryptic genetic variation
Source: PLoS Genet. 2020 May 11;16(5):e1008801. doi: 10.1371/journal.pgen.1008801 (PMC7241848; doi:10.1371/journal.pgen.1008801)

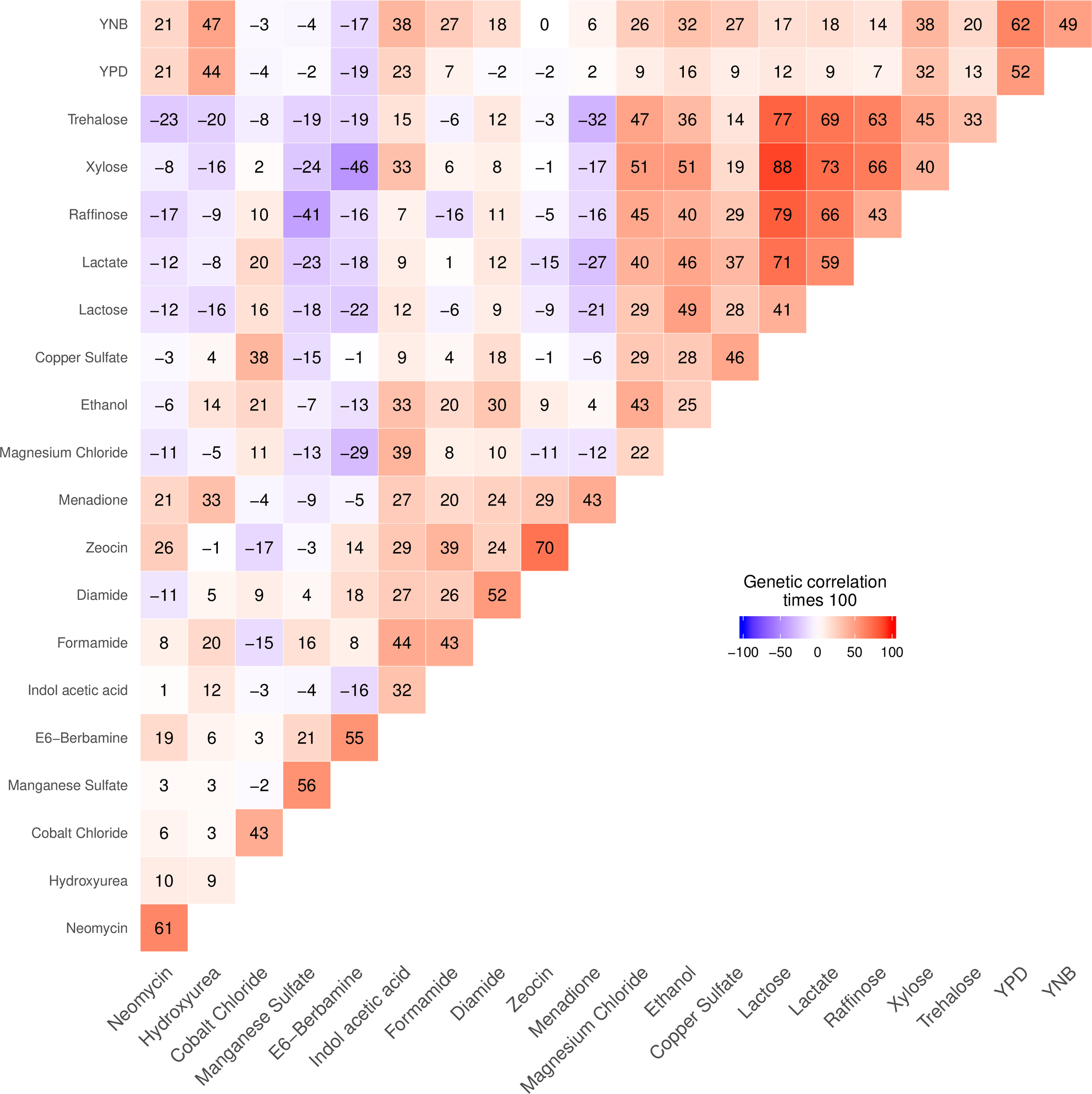

Supplement: S1 Fig — Numbers in the cells are 100 times the genetic correlation, and environments were sorted based on their order after hierarchical clustering. (TIF) [file pgen.1008801.s001.tif]

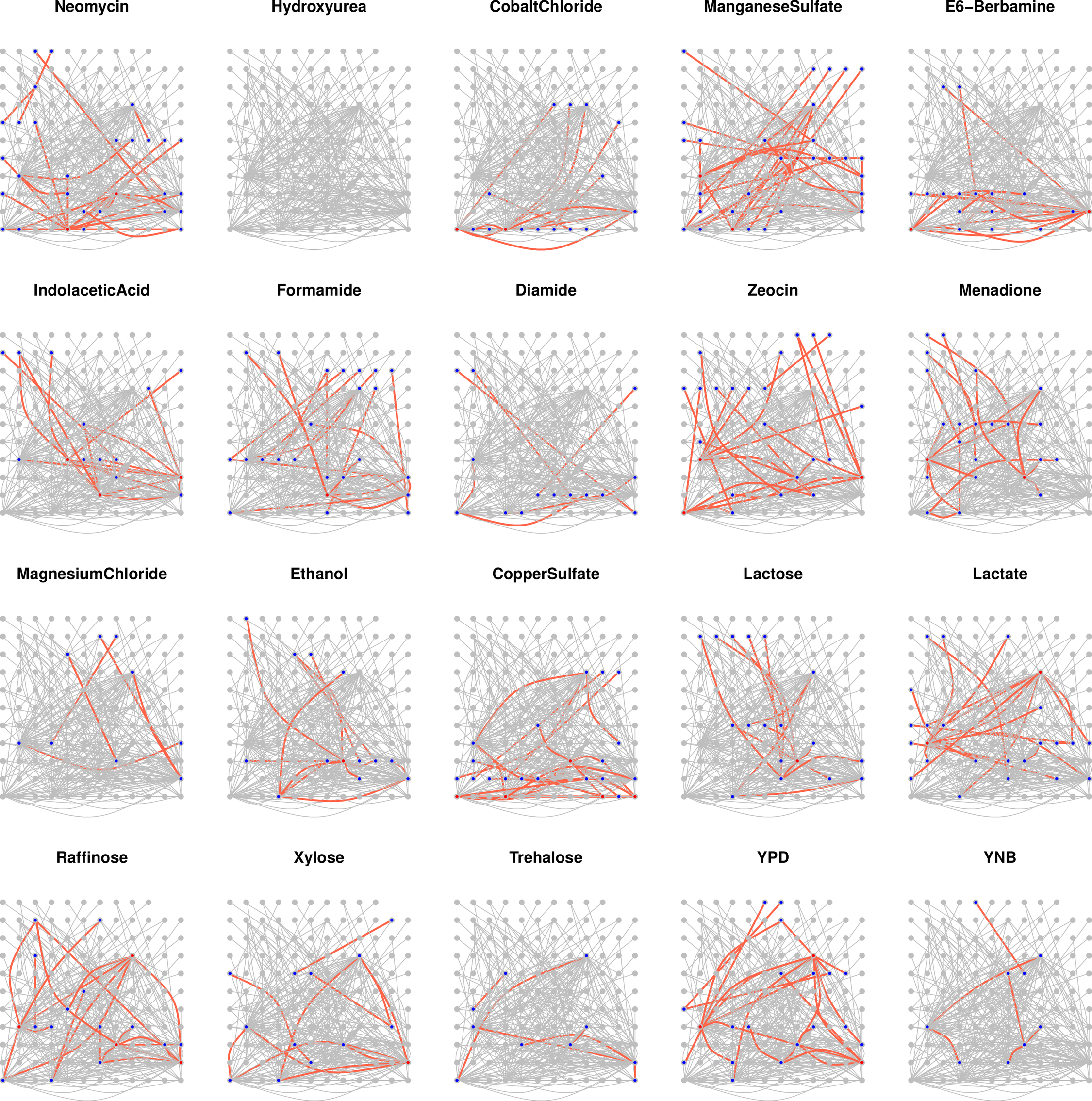

Supplement: S2 Fig — Each dot in this plot is an epistatic QTL and the colour of the dot describes if the locus is detected with epistatic interaction for the current media with yes being blue or red (connected with more than 4 other loci) and no being grey. The pairwise interactions between loci are indicted by connected edges. The number of edges connecting two loci describe the number of times it is detected across 20 mediums, and the detected connection for current medium is highlighted with red (detected in Bloom et al,) and grey (other medias). (TIF) [file pgen.1008801.s002.tif]

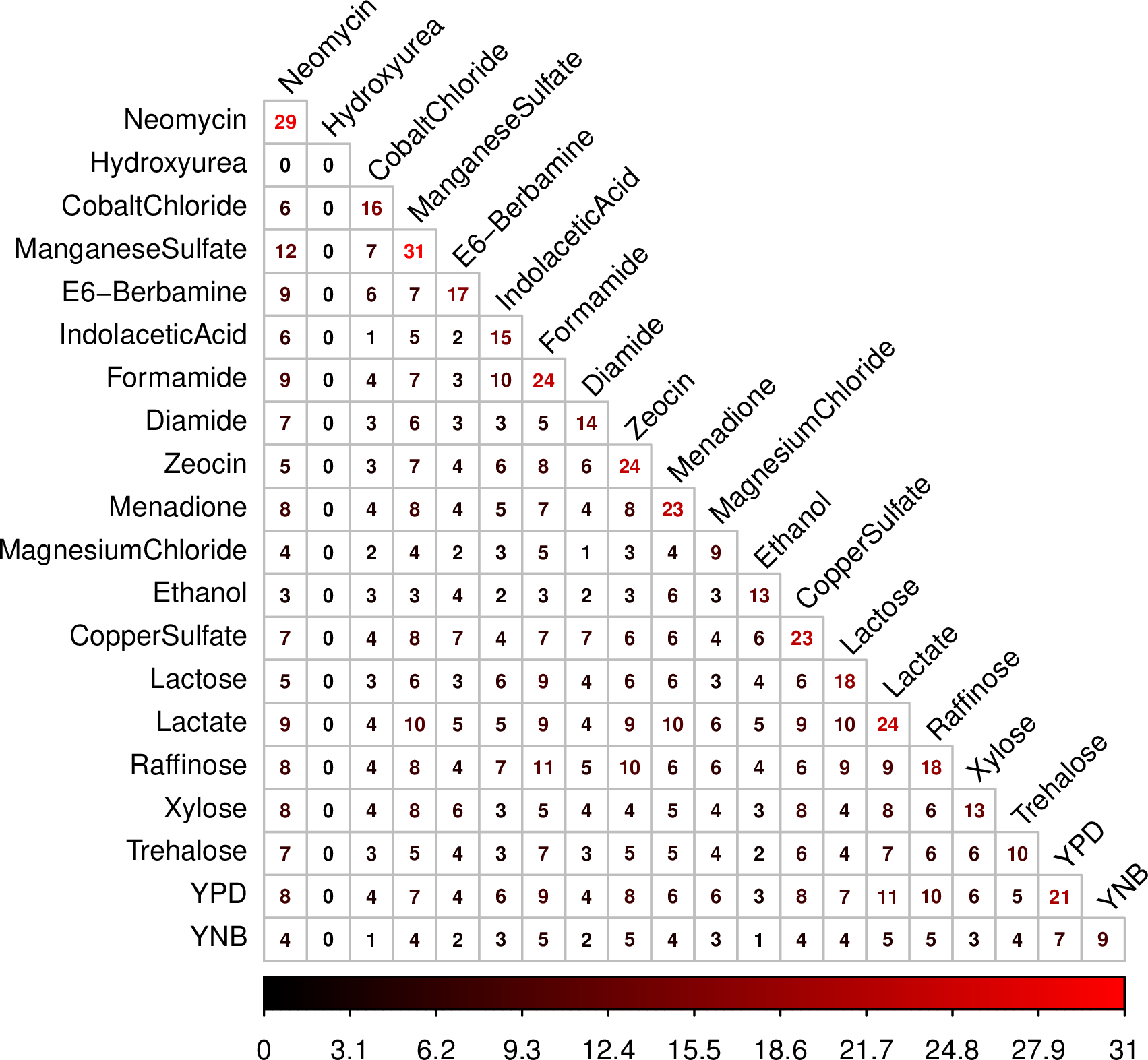

Supplement: S3 Fig — Numbers on the diagonal are the number of epistatic loci detected in a particular environment, and numbers in the cells are the number of overlapping epistatic loci between the pairs of environments. Phenotypes are sorted based on their order after hierarchical clustering. (TIF) [file pgen.1008801.s003.tif]

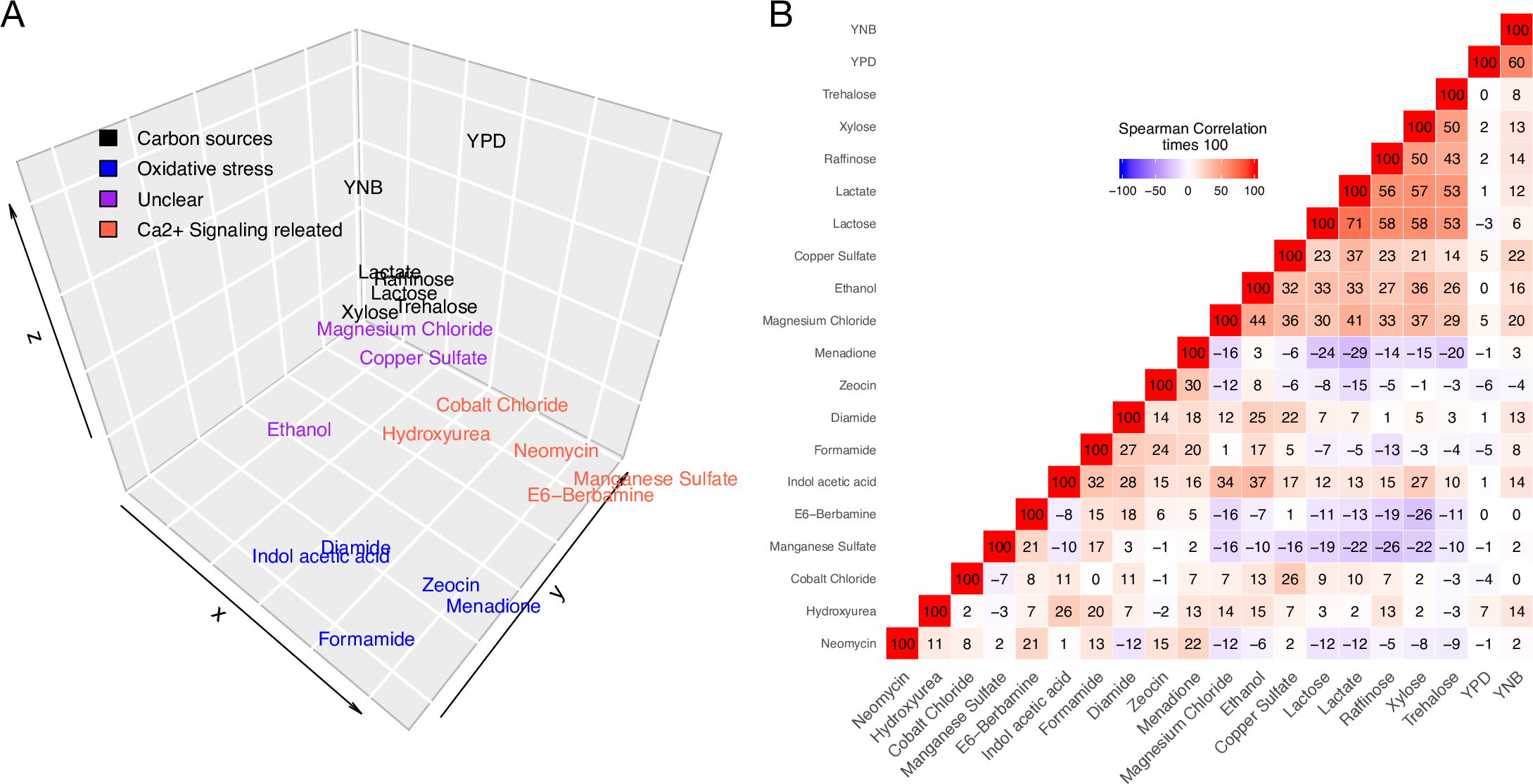

Supplement: S4 Fig — A). 3-dimentainal PCA plot of the yeast growth measured as the radial of colony on 20 different mediums. These mediums were made by adding small-chemical molelues to mimic different enviroments [30]. B). Pairwise Spearman rank correlation among growth measured on these 20 mediums. Numbers in the cell are 100 times the Spearman correation, and environments were sorted based on their order after hierarchical cluster. (TIF) [file pgen.1008801.s004.tif]

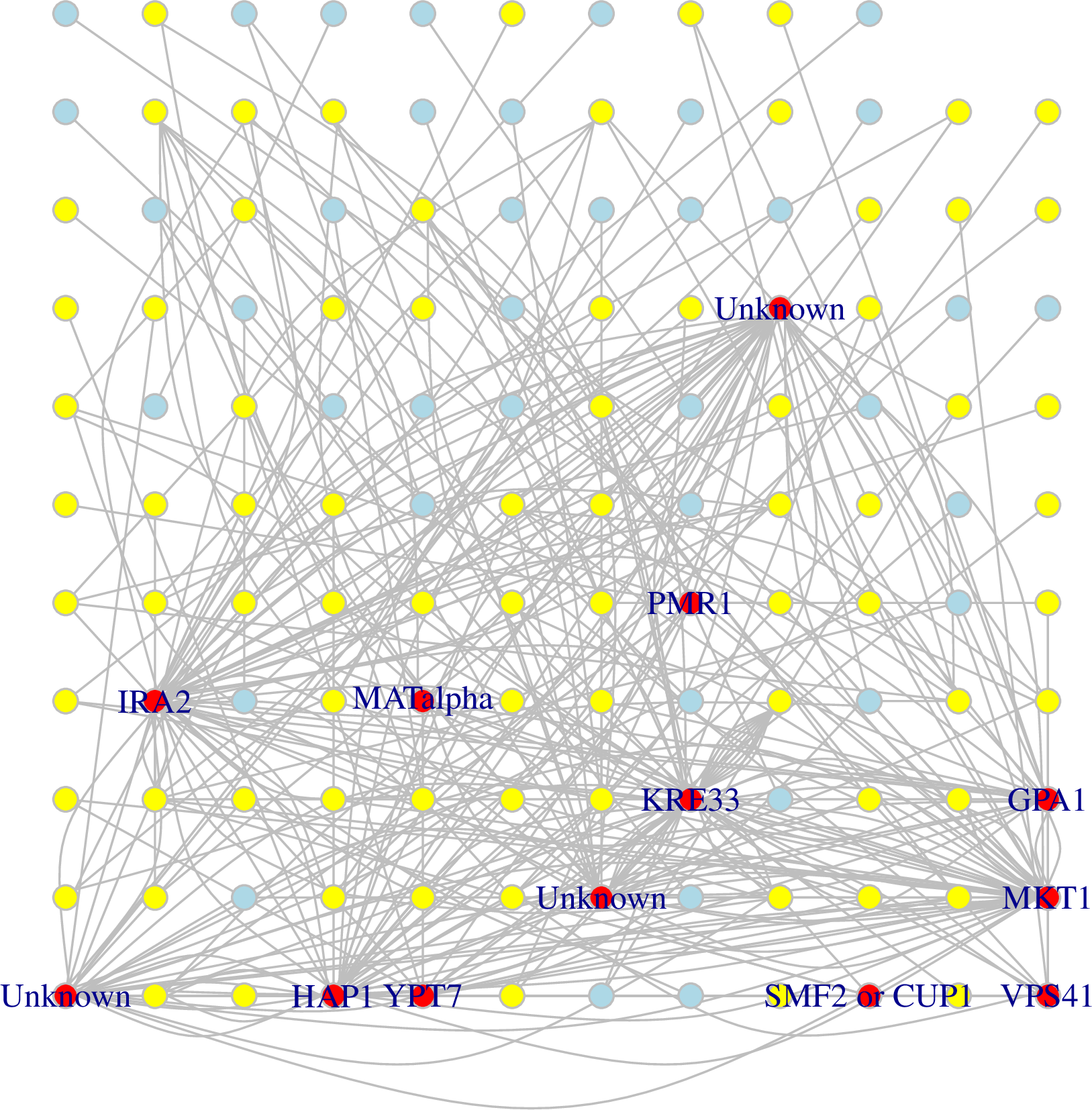

Supplement: S5 Fig — 13 hubs connected with more than 4 loci in at least 1 environment is highlighted in red, loci epistatically interact with these hubs in at least one environment are labeled in yellow. (TIF) [file pgen.1008801.s005.tif]

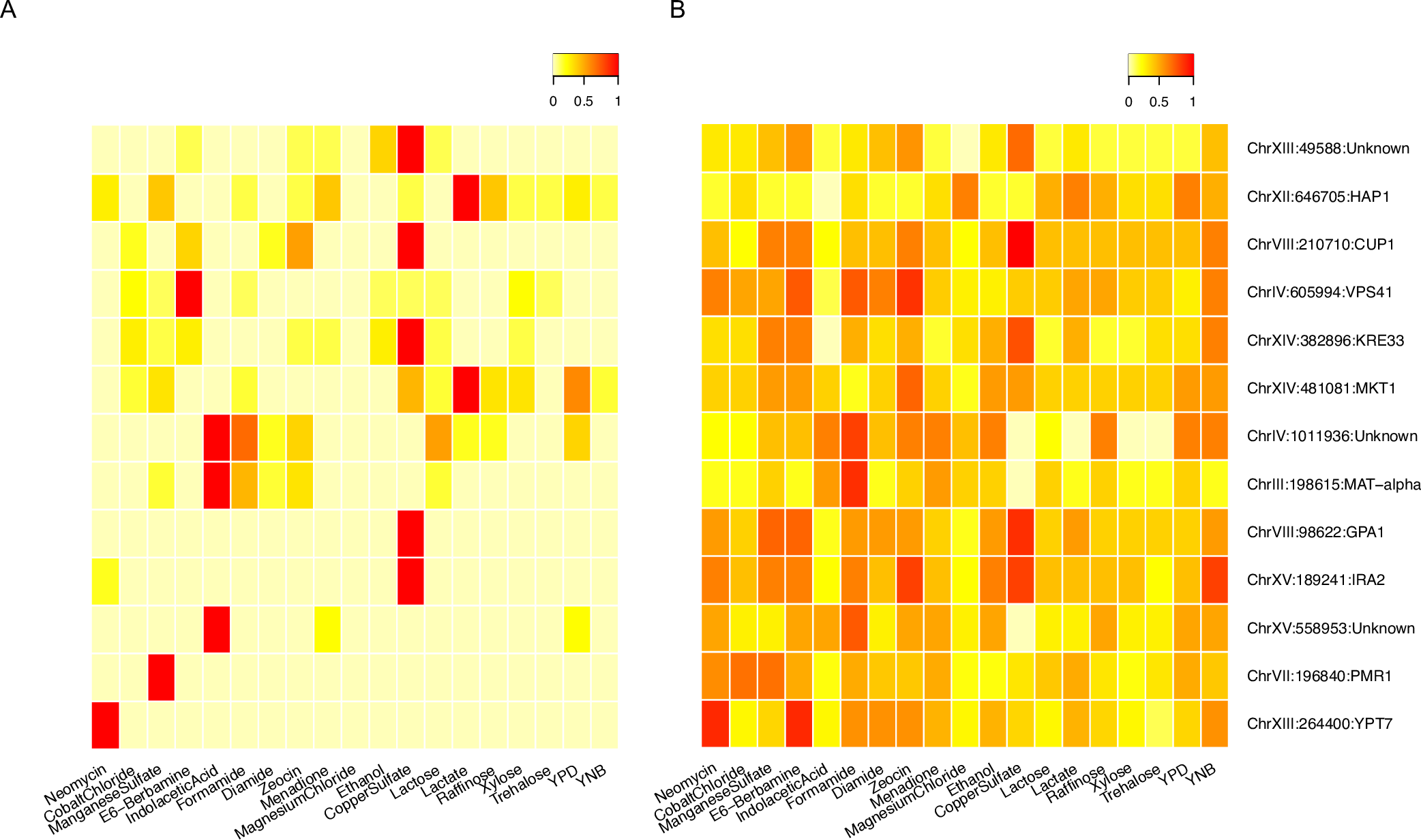

Supplement: S6 Fig — In total, 13 epistatic networks were defined across 20 environments (represented in each column). Each row represents the activity of interactions in a particular network with corresponding hub alleles and candidate genes marked to the left. The colour intensity illustrate the proportion of loci, defined by their hub-QTL in a particular environment, that are connected as epistatic QTL (A) or additive QTL(B). The environments are sorted based on their order after hierarchical cluster. (TIF) [file pgen.1008801.s006.tif]

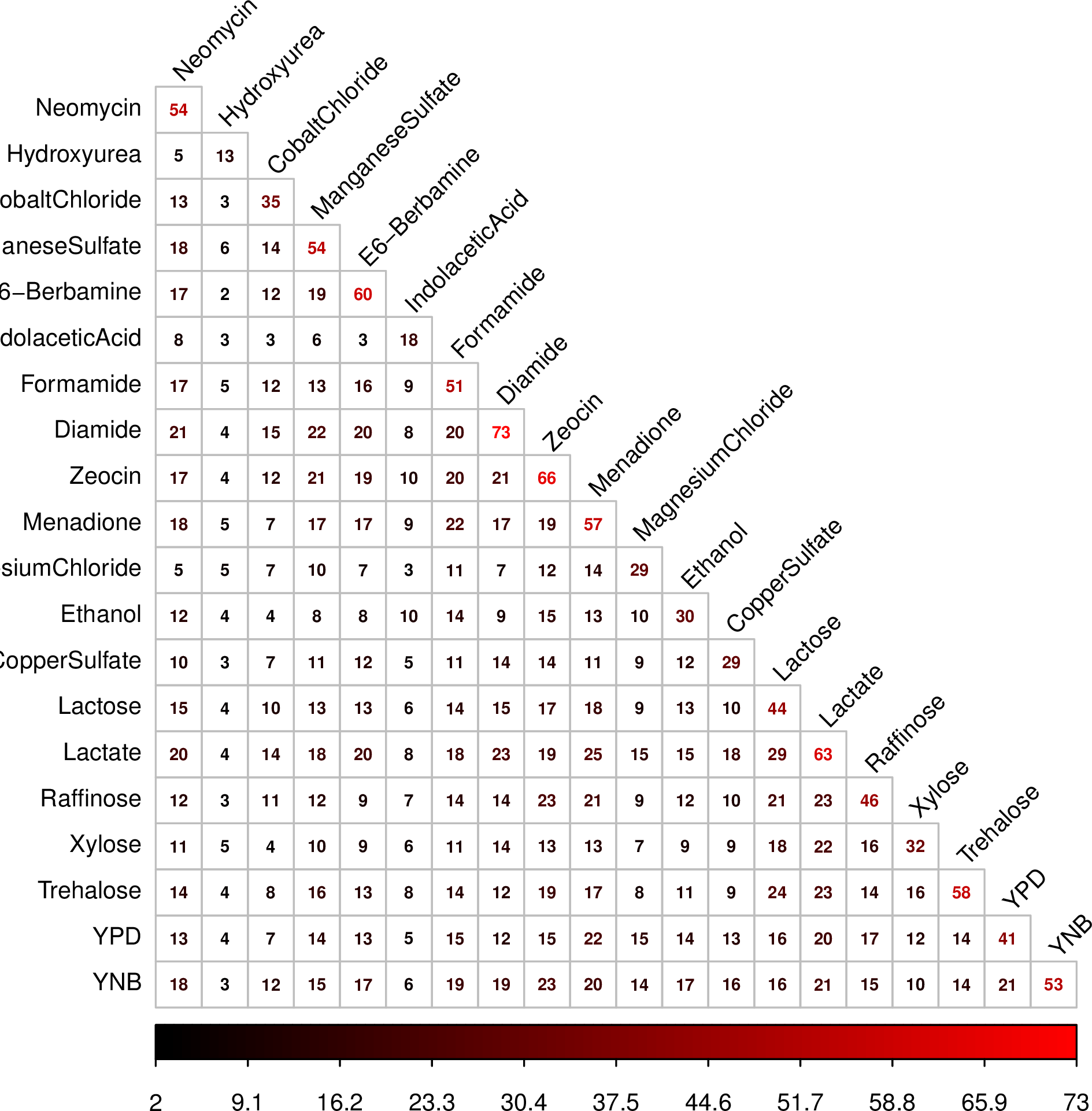

Supplement: S7 Fig — Numbers on the diagonal are the numbers of additive loci detected for a particular enviroment, and numbers in the cell are the number of overlap addtive loci. Phenotype were sorted based on their order after hierarchical cluster. (TIF) [file pgen.1008801.s007.tif]

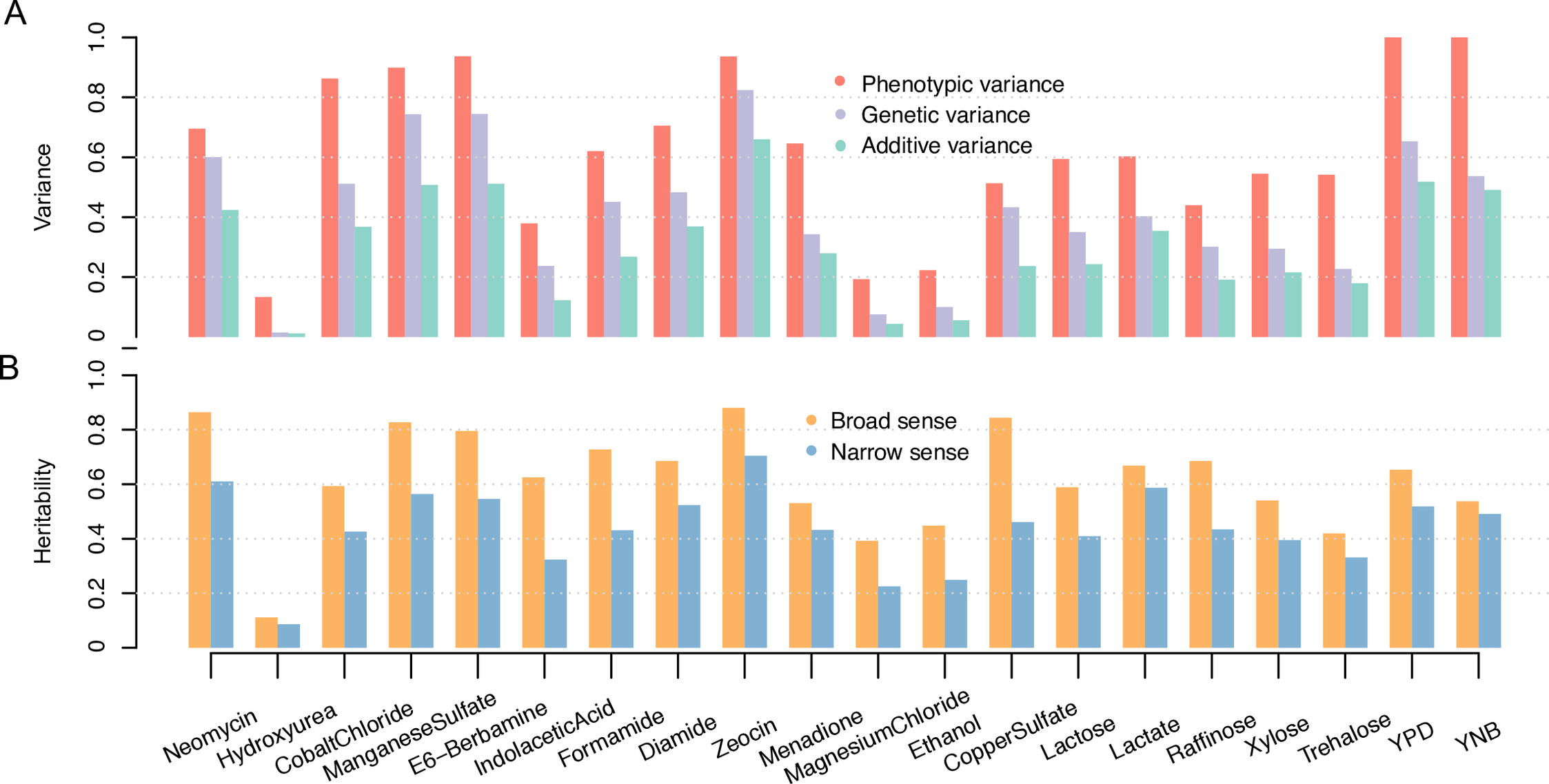

Supplement: S8 Fig — A). The phenotypic-, total genetic- and additive genetic variances for growth on 20 growth media. Total- and additive genetic variances were estimated as the product of the phentypic variance and the broad-/narrow-sense heritabilities, respectively, (panel B) from Bloom et al [30]. (TIF) [file pgen.1008801.s008.tif]

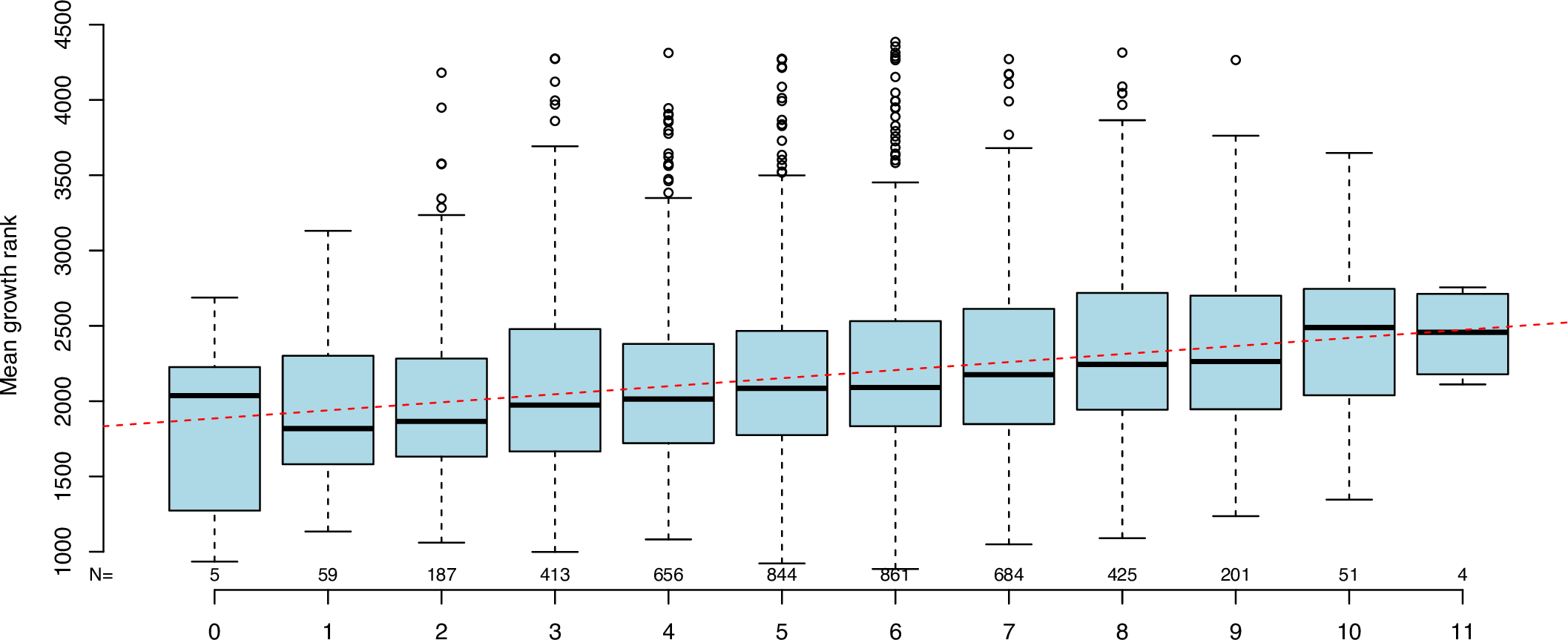

Supplement: S9 Fig — X-axis is the number of non-compacitated alleles across 13 hubs detected in our study, and y-axis is the mean growth rank obtained by first rank the growth measurements across 20 enviroemtns and then taking the artihmatical mean. (TIF) [file pgen.1008801.s009.tif]
