# Supplementary material for: Dynamic genetic architecture of yeast response to environmental perturbation shed light on origin of cryptic genetic variation
Source: PLoS Genet. 2020 May 11;16(5):e1008801. doi: 10.1371/journal.pgen.1008801 (PMC7241848; doi:10.1371/journal.pgen.1008801)
Supplement: S1 Note — (PDF) [file pgen.1008801.s012.pdf]

Supporting note 1

Evaluation of the independence of the 130 loci

In a F2 cross, fitting 130 loci in the same model raise the concern that they may have lost their independence and the fraction of additive variance may have asymptoted with a smaller collection of loci and fitting 130 loci might be equivalent to barcoding the individuals, resulting in the estimated additive variance biased upwards.

To evaluate this, we calculated the cumulative variance explained by increasing the number of loci modelled for each of the analysed traits. As shown in Figure N1 it plateaus (reaches an asymptote), and this often after including between 20-50 loci in the model.

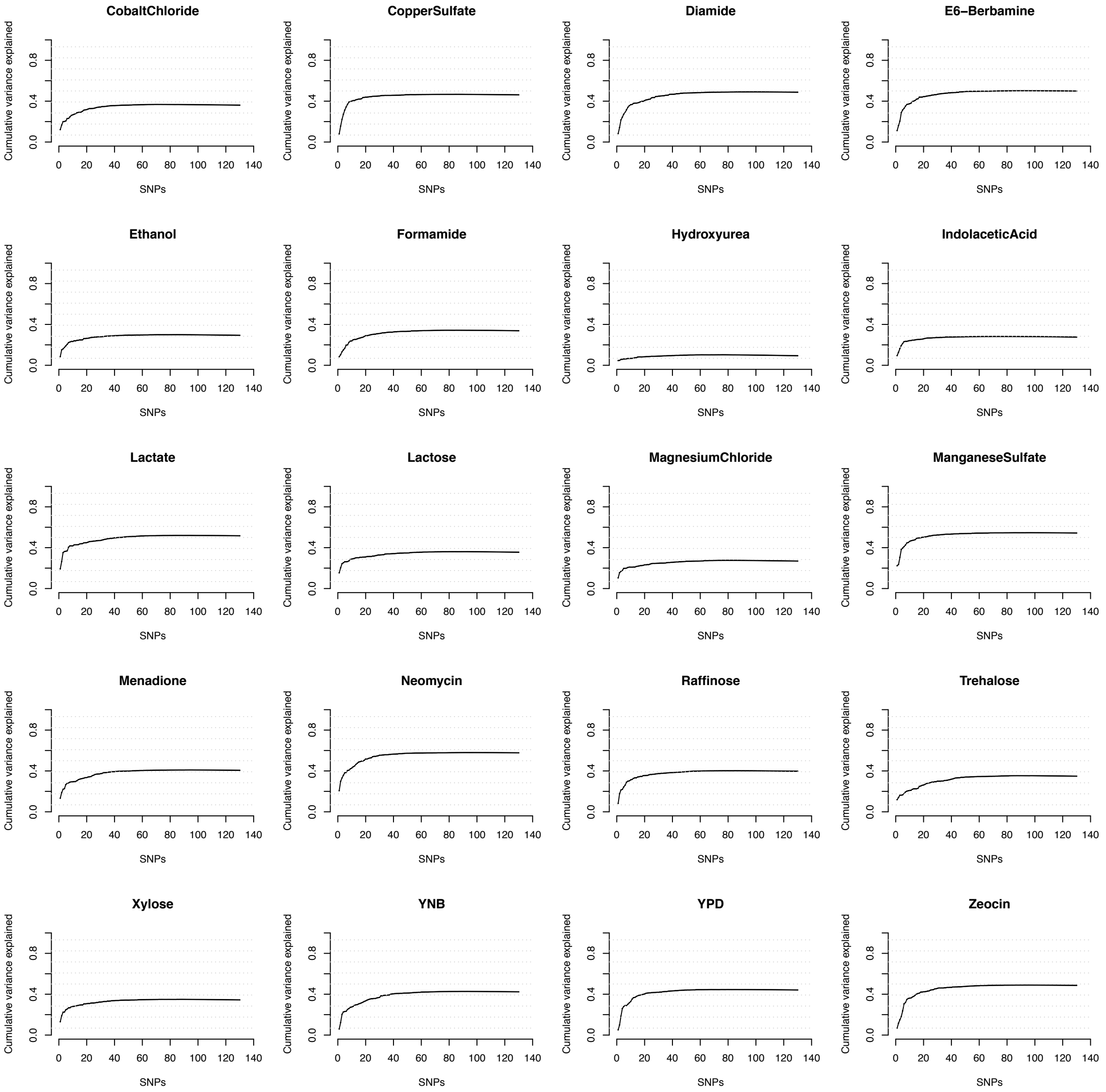

Figure N1. **Cumulative variance explained by adding one of the 130 loci at a time to the model in the order determined by increasing estimated effect sizes.**

However, such an asymptote could occur for several reasons. One would be that it is not possible to map 130 independent loci in this population. Then one would instead start to model the individual segregants ('barcoding') in this way. Another, suggested by earlier mapping work in this population, is that only a fraction of the growth QTL contributes in each environment and therefore the rest contribute little (if any) phenotypic variance there. We evaluated this further in two ways.

First, we simulated 130 equal-size QTL at the locations of those QTL included in this study, and then estimated the cumulative variance explained by these after including them one at the time to the model. The results show that the cumulative variance explained continues to increase until the last simulated locus is added (Figure N2). This indicates that the observed plateaus at 20-50 loci is unlikely due to lack of independence between the modelled loci in this population.

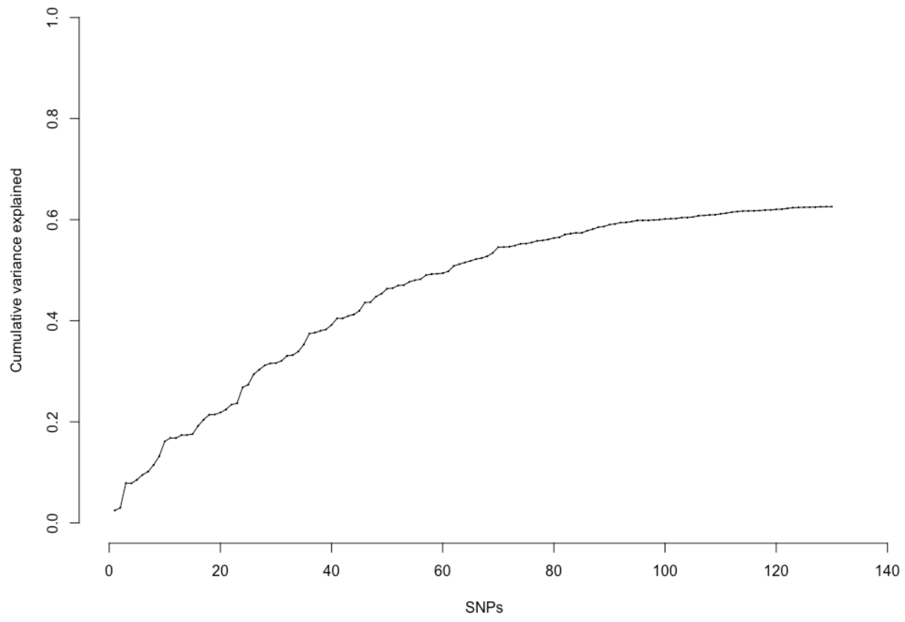

Figure N2. Cumulative variance explained in simulated data. Genotypes at 130 simulated QTL were assigned equal effects and a random residual was added to obtain the phenotype. *The cumulative variance explained by adding these one at a time to the model is shown with a line.*

Second, we evaluated the “barcoding” hypothesis directly using simulations. This is achieved by exploring how much variance the genotypes at 100 random draws of 130 selected loci could capture for each of the studied traits. As illustrated in Figure N3, for most of the traits the 130 QTL in the network capture more variance than any of the extremes in the random sets generated in the simulations.

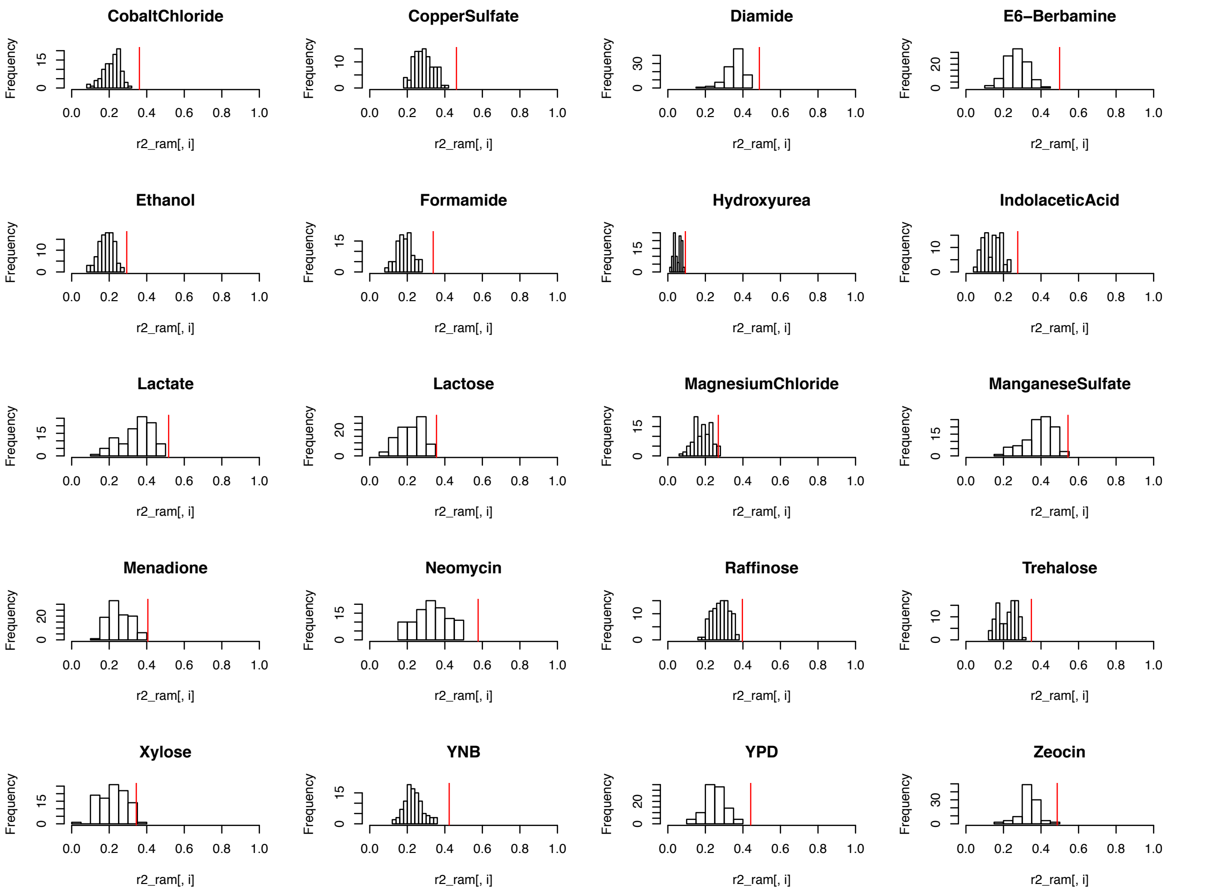

Figure N3. Histograms illustrating the amount of variance explained by 100 random sets of 130 loci in relation to that explained by the 130 QTL in the evaluated network (red vertical line).

These analyses cannot completely rule out the possibility that by including all 130 loci in the model, some of them will have lost their independence. However, the simulations illustrate that it is unlikely to be so. Rather, the data seems sufficiently powerful to allow modelling of this many loci and the observed plateaus/asymptotes in the cumulative variances explained are rather due to only subsets of loci being active in each environment. Given that the results to not clearly support that including this number of loci leads to results equivalent to bar-coding/”individual segregant” modelling, we did not explicitly discuss this in the revision. However, if we missed something or the reviewer/editor feel that it should be discussed

regardless, we are open to reconsider this and revise based on your further comments/suggestions.
