# Supplementary material for: Dynamic genetic architecture of yeast response to environmental perturbation shed light on origin of cryptic genetic variation
Source: PLoS Genet. 2020 May 11;16(5):e1008801. doi: 10.1371/journal.pgen.1008801 (PMC7241848; doi:10.1371/journal.pgen.1008801)
Supplement: Attachment — Submitted filename: plos-genetics-review.pdf [file pgen.1008801.s014.pdf]

As a reviewer I still have major concerns about the appropriateness of the analyses presented in this manuscript. My concerns are both biological and methodological as I'll describe below. I will try not to be "overly-aggressive" in my framing of these concerns, but I feel it necessary to the highlight what I see as a key methodological flaw. I will also note that at the same time I am quite positive about the questions this manuscript sets out to address.

My key biological concern can be summarized as "what is a trait?" For an analysis of Genotype-by-Environment interactions to make sense, the underlying assumption is that one has measured the same trait under different environmental conditions. Here the trait is ostensibly "growth". However, my concern is that the biological underpinnings of yeast growth in the different treatments is different enough that many of the subsets of "growth" measures should be treated as different traits, absent an analysis that convincingly demonstrates them to share a high degree of similarity (see below for suggestions about one such quantitative model for approaching this).

While there may be a "common growth factor" that is shared across the different measures, I argue that for many of the traits the "condition specific factors" may largely outweigh such a common factor. If this is the case, then the fact that the inferred G-by-E interactions differ across treatments is not surprising but is simply a consequence of the fact that the authors are estimating the genetic architecture of what in essence are many different traits.

In order to make this argument quantitative, I ask the authors (and Editor) to consider the path analysis/SEM style model below (see embedded image). In this figure, the "Common Growth Factor" and "Shared Growth Factors" represent latent variables that contribute to the variation of each of the individual traits (growth in each of the environments). The unlabeled arrows represent trait specific contributions to each growth trait. An SEM style model like this posits that correlation between any two pairs of traits is a function of the "Common Growth Factor" and any "Shared Growth Factors" that the traits may have in common. Note that models like this are common in the morphometric literature, where the "Common Growth Factor" would be usually replaced by a "General size factor" (e.g. Bookstein, F. L. 1991. Morphometric Tools for Landmark Data. Geometry and Biology. Cambridge University Press: New York).

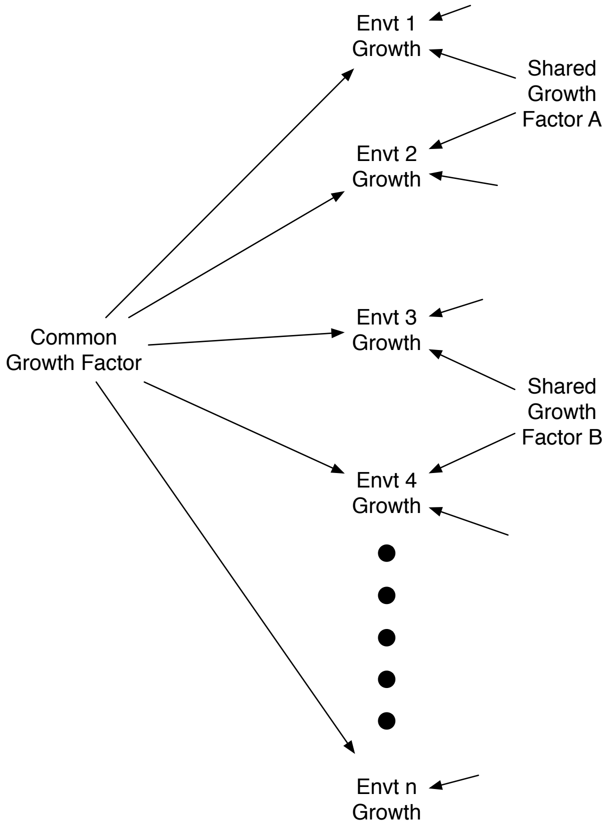

I suggest that estimating such a model, analyzing the contribution of this "Common Growth Factor" to the variance of each traits, and then comparing the genetic architecture of this "Common Growth Factor" across treatments would be both more biologically and statistically sound approach to analyzing G-by-E interactions. I predict (though can't say for sure not having done the analysis) that an analysis that reflected both the biological complexity of the traits at hand as well as the statistical complexity that describes their covariance structure would lead to greater biological interpretability of the results.
