# Supplementary material for: Dynamic genetic architecture of yeast response to environmental perturbation shed light on origin of cryptic genetic variation
Source: PLoS Genet. 2020 May 11;16(5):e1008801. doi: 10.1371/journal.pgen.1008801 (PMC7241848; doi:10.1371/journal.pgen.1008801)
Supplement: Attachment — Submitted filename: Response-to-reviewer_rev1.docx [file pgen.1008801.s013.docx]

Dear Editor,

First, we would like to thank you and the three reviewers for the constructive comments that were useful for improving the manuscript. It has now been revised with edits throughout, with the major changes in the discussion. We believe that these and the detailed responses to the reviewer/editorial comments, address all concerns raised by you and the external reviewers.

A detailed response to all the comments, including a description how they have been implemented in the revision, is attached. We hope you find that our responses and edits to the manuscript now make it suitable for publication. If you have any further questions, comments or concerns, we would be happy to answer these as well.

Best regards,

Yanjun Zan and Örjan Carlborg

Dear Dr zan,

*Thank you very much for submitting your Research Article entitled 'Dynamic genetic architecture of yeast growth response to environmental perturbation shed light on origin of hidden genetic variation' to PLOS Genetics. Your manuscript was fully evaluated at the editorial level and by independent peer reviewers. The reviewers appreciated the attention to an important topic but identified some aspects of the manuscript that should be improved.*

*All three reviewers are highly qualified to evaluate the manuscript.  Nonetheless, whereas Reviewers #1 and #3 were quite positive, Reviewer #2 was significantly more skeptical.*

*Q1: Reviewer #2’s primary concern is that growth was measured by colony radius which is a phenotype that may have multiple confounding inputs.  This is a reasonable question.  As an aside, the editors noted that the tone of these comments was at times overly-aggressive, but that tone did not influence our assessment of the underlying scientific/technical issues. Can you use an independent measurement to validate the robustness of colony radius as a measure of growth in your experiments?*

R1: Yes, we agree that this question is reasonable. And we would, if possible, have been very interested in comparing how robust our results were to the way of measuring growth. However, as a computational lab we are unable to perform such experiments by ourselves as we rely on external public data for this work. Our hope is that the publication of this work will motivate experimental scientists to make this kind of validations, for example by addressing how well measurements from solid and liquid media are correlated. A previous study [1] has reported that growth on solid and liquid media may only resemble each other during the initial growth stage and that there are significant differences thereafter. Such studies might thus be valuable to gain further insights to G by G by E interactions by highlighting other aspects of growth that is not captured when measured as colony size.

*Q2: Barring that, I think it would be important to explicitly discuss this caveat when discussing the conclusions of the manuscript.*

R2: This is now discussed in the revised manuscript.

*Q3: We therefore ask you to modify the manuscript according to the review recommendations before we can consider your manuscript for acceptance. Your revisions should address the specific points made by each reviewer.*

R3: The manuscript has now been revised following the recommendations of the editor and reviewers as detailed in the responses below.

*In addition, we ask that you:*

*Q4: 1) Provide a detailed list of your responses to the review comments and a description of the changes you have made in the manuscript.*

R4: Detailed responses to the review comments are included together with a manuscript version where the changes are highlighted.

*Q5: 2) Upload a Striking Image with a corresponding caption to accompany your manuscript if one is available (either a new image or an existing one from within your manuscript). If this image is judged to be suitable, it may be featured on our website. Images should ideally be high resolution, eye-catching, single panel square images. For examples, please browse our* [*archive*](http://www.plosgenetics.org/article/browse/volume)*. If your image is from someone other than yourself, please ensure that the artist has read and agreed to the terms and conditions of the Creative Commons Attribution License. Note: we cannot publish copyrighted images.*

R5: Unfortunately, we do not have any striking image relating to this work.

*We hope to receive your revised manuscript within the next 30 days. If you anticipate any delay in its return, we would ask you to let us know the expected resubmission date by email to* [*plosgenetics@plos.org*](mailto:plosgenetics@plos.org)*.*

*If present, accompanying reviewer attachments should be included with this email; please notify the journal office if any appear to be missing. They will also be available for download from the link below. You can use this link to log into the system when you are ready to submit a revised version, having first consulted our* [*Submission Checklist*](http://journals.plos.org/plosgenetics/s/submit-now#loc-submission-checklist)*.*

*While revising your submission, please upload your figure files to the* [*Preflight Analysis and Conversion Engine*](http://pace.apexcovantage.com/) *(PACE) digital diagnostic tool. PACE helps ensure that figures meet PLOS requirements. To use PACE, you must first register as a user. Then, login and navigate to the UPLOAD tab, where you will find detailed instructions on how to use the tool. If you encounter any issues or have any questions when using PACE, please email us at* [*figures@plos.org*](mailto:figures@plos.org)*.*

*Q6: Please be aware that our* [*data availability policy*](http://journals.plos.org/plosgenetics/s/data-availability) *requires that all numerical data underlying graphs or summary statistics are included with the submission, and you will need to provide this upon resubmission if not already present. In addition, we do not permit the inclusion of phrases such as "data not shown" or "unpublished results" in manuscripts. All points should be backed up by data provided with the submission.*

R6: All scripts used for the analyses, as well as raw and intermediate numerical data used to generate the figures, are provided in GitHub <https://github.com/yanjunzan/Yeast-GGE>. This information is now included in the revised manuscript.

*PLOS has incorporated* [*Similarity Check*](http://www.crossref.org/crosscheck.html)*, powered by iThenticate, into its journal-wide submission system in order to screen submitted content for originality before publication. Each PLOS journal undertakes screening on a proportion of submitted articles. You will be contacted if needed following the screening process.*

*To resubmit, you will need to go to the link below and 'Revise Submission' in the 'Submissions Needing Revision' folder.*

*Please let us know if you have any questions while making these revisions.*

*Yours sincerely,*

*Gregory P. Copenhaver*

*Editor-in-Chief*

*PLOS Genetics*

**Detailed responses to Reviewer's Questions**

***Reviewer #1:***

*In this manuscript, the authors reanalyze a large existing dataset to work to better understand the potential role of epistasis in influence quantitative genetic variation in growth across a number of environments in yeast. This contributes to the growing level of data showing that GxGxE has an underappreciated contribution to quantitative traits. I only have a few suggestions.*

*Q1: In Figure 2E, do the authors have an estimate on how the additive variance explained responds to decreasing the number of loci? I ask because with 130 loci and the number of progeny utilized, the loci may have lost their independence and the fraction of additive variance may have asymptoted with a smaller collection of loci. Essentially, using 130 loci may be the equivalent of bar coding each individual and thus the additive estimate is functioning as a broad sense estimate at this level. Some discussion on this possibility would help the reader who is disinclined from epistasis to get past a high additive explanatory potential.*

R1: This is an interesting point that we have now evaluated in further detail. First, we calculated the cumulative variance explained by increasing the number of loci modelled for each of the analysed traits. As shown in Figure 1 it plateaus (reaches an asymptote), as suggested by the reviewer, and this often after including between 20-50 loci in the model.

***Figure 1****. Cumulative variance explained by adding one of the 130 loci at a time to the model in the order determined by increasing estimated effect sizes.*

However, such an asymptote could occur for several reasons. One, as suggested by this reviewer, would be that it is not possible to map 130 independent loci in this population. Then one would instead start to model the individual segregants (‘barcoding’) in this way. Another, suggested by earlier mapping work in this population, is that only a fraction of the growth QTL contributes in each environment and therefore the rest contribute little (if any) phenotypic variance there. We evaluated this further in two ways.

First, we simulated 130 equal-size QTL at the locations of those QTL included in this study, and then estimated the cumulative variance explained by these after including them one at the time to the model. The results show that the cumulative variance explained continues to increase until the last simulated locus is added (Figure 2). This indicates that the observed plateaus at 20-50 loci is unlikely due to lack of independence between the modelled loci in this population.

***Figure 2****. Cumulative variance explained in simulated data. Genotypes at 130 simulated QTL were assigned equal effects and a random residual was added to obtain the phenotype. The cumulative variance explained by adding these one at a time to the model is shown with a line.*

Second, we evaluated the “barcoding” hypothesis directly using simulations. This by exploring how much variance the genotypes at 100 random draws of 130 selected loci could capture for each of the studied traits. As illustrated in Figure 3, for most of the traits the 130 QTL in the network capture more variance than any of the extremes in the random sets generated in the simulations.

***Figure 3****. Histograms illustrating the amount of variance explained by 100 random sets of 130 loci in relation to that explained by the 130 QTL in the evaluated network (red vertical line).*

These analyses cannot completely rule out the possibility that by including all 130 loci in the model, some of them will have lost their independence. However, the simulations illustrate that it is unlikely to be so. Rather, the data seems sufficiently powerful to allow modelling of this many loci and the observed plateaus/asymptotes in the cumulative variances explained are rather due to only subsets of loci being active in each environment. Given that the results to not clearly support that including this number of loci leads to results equivalent to bar-coding/”individual segregant” modelling, we did not explicitly discuss this in the revision. However, if we missed something or the reviewer/editor feel that it should be discussed regardless, we are open to reconsider this and revise based on your further comments/suggestions.

*Q2: Is it that the environment is rewiring the network or altering how the output of the network is displayed? Rewiring implies (not inherently the authors intended implication) that the molecular connections are changing. I might suggest a different non-molecular wording.*

R2: Thank you for this helpful advice! We do, indeed, not intend to suggest that the molecular connections change but rather (as suggested) that the (phenotypic) output does. It has been difficult to find an appropriate term to describe this and have now tried to revise in spirit of this suggestion (See changes marked in the revised manuscript). We ourselves feel that it has improved clarity, but are open to further changes based on additional suggestions.

*Q3: Am I correct in interpreting Figure 5A as implying that the idea of rare alleles tending to have large effects needs to be tempered to say that they might also tend to be more environmentally and epistatically dependent? If that is correct, is there a reason to at best only provide veiled allusions to this outcome?*

R3: With the limitation that what is analysed here is an experimental cross where all loci have allele frequencies of 0.5, we agree with this interpretation: large effect alleles, that often are believed to be rare in segregating populations, might in fact be more common as their effects could also be moderated by epistasis and environment. This was not discussed in the original manuscript as it could be considered too speculative, but following this comment we have added it in the revision.

*Editorial comments*

*Q4: As a personal thought, I tend to prefer cryptic genetic variance as crypsis can be a passive process while hidden implies effort/mechanism to hide. I know that the community has not settled on a nomenclature so I won’t attempt to enforce one.*

R4: Thank you for your suggestion. We have updated the manuscript to primarily use cryptic genetic variance.

*Q5: Line 67, I believe the authors mean E. coli, S. cerevisiae and A. thaliana as at least one of the ensuing manuscripts is in Arabidopsis. And I do believe that the plant citation works to investigate some of the quant gen parameters discussed in line 91-94. Albeit in far narrower GxGxE collection.*

R5. Thank you for your suggestions, these have now been implemented in the revision.

*Q6: If I’m reading correctly, all the multi-locus epistasis citations are in yeast. There are some papers on higher order epistasis in Plants and Drosophila that would help to move the conclusions beyond yeast.*

R6: Thank you for highlighting this, we have added relevant references from *D. melanogaster* and *A. thaliana* in the revised manuscript and updated the text accordingly.

*Q7: In Line 420, I’m struggling with this equation Vtotal= H2 *Vp = H2. Is this correct as it seems odd to say broad sense heritability times Vp is equal to broad sense heritability. What am I missing?*

R7: Upon further thought, we realize that this is confusing. Our intention was merely to illustrate (with a formula) that the amount of total genetic variance is equal to the broad sense heritability (H^2^) times the phenotypic variance (V_p_). Since the phenotypic variance was standardised to variance 1 (V_p_ =1), the total genetic variance is here actually equal to the estimated broad sense heritability. This formula is, however, not necessary and likely to cause more confusion than clarity. It has therefore now been removed and replaced with a description in text instead. We hope that this description clear and are open to discuss this further if this has not improved clarity.

**Reviewer #2:**

*Q1: This ms. aims to examine epistatic (GxG) and genotype-by-environment (GxE) interactions using a previously published data set (Bloom et al. 2015) consisting of large mapping population of segregants in the budding yeast S. cerevisiae whose growth was measured under a large number of environmental conditions and stressors. The novel angle the authors employ, relative to two earlier studies using the same dataset (Bloom et al., Forsberg et al. 2017), is to treat growth as a single complex trait, rather than distinct traits for each environment, and use this single trait to compare "reorganization" of epistatic interactions across environments. Unfortunately, while this is a clever idea in the abstract, the biological reality of endpoint growth measures (the trait measured in the original Bloom et al. study) makes this highly questionable in practice, and calls into question whether any useful biological insights are likely to emerge from such an analysis.*

R1: We agree with the reviewer that it would have been very valuable to have more detailed phenotypes available to obtain more precise molecular insights into the specific underlying biological processes involved. Limited by the fact that the current dataset was restricted to endpoint growth measures, we performed a quantitative genetics analysis of the data. Consequently, this was not an attempt to provide biological (functional/molecular) insights to the detailed contribution by specific pathways/networks to the trait. Rather it was an attempt to use a statistical genetics modeling approach to advance our general understanding on the contributions from G by G by E interactions to complex trait variation and their influence on metrics commonly used in such work in higher organisms. Despite the lack of molecular data, valuable insights were provided from this study for the statistical/quantitative genetics community. Our hope is that also some molecular microbial geneticists will find the work sufficiently interesting to take the results from this further and do the necessary work to study the biology of some networks, and potentially underlying biological pathways. Such follow-up studies would be extremely valuable to broaden our understanding on how changes in genetic background and environment contribute to altered biological processes involved in yeast growth.

*Q2: Bloom and co-authors simply measured colony radii for each segregants after 48 hrs of growth on various agar plates. Colony radius reflects a mix of various cell biological and physiological traits -- division rate, cell size and cell shape, cell-cell interactions, etc. While various measures of growth on agar plates is often the microbiologists primary screening tool, no careful microbial geneticist would presume a priori that growth under neomycin stress is comparable to growth in xylose (to take as an example two of the more physiologically distinct conditions in the Bloom et al. data). While I don't doubt that some of the subsets of environments in the data affect underlying signaling and regulatory networks in similar ways, and hence would in some sense be comparable traits, this seems like a hypothesis to be tested not taken a priori to be true.*

R2: We fully agree that one cannot take for granted that growth under one type of stress is regulated by the same genes, networks and pathways as those under another type of stress. In fact, one of the aims of this study was to explore this by evaluating to what degree the statistically mapped loci and networks overlap across different growth conditions. Since specific networks/pathways were not approached, but rather a genome-wide approach was taken focusing on overlaps of trait associated loci, no assumptions regarding biological/functional overlaps are necessary beforehand, but rather the analysis will provide results that makes it possible to explore whether such overlaps are likely to be found in later molecular follow-up studies. Actually, it does seem like even in highly physiologically distinct conditions, there is a set of core loci contributing to growth. Taking the same example as the reviewer raised, we found a considerable overlap in the loci contributing to growth (as defined in the assays by Bloom et al) for Neomycin and Xylose, in that 12/48 additive (and 8/18 epistatic) Xylose loci overlap with loci detected for growth on Neomycin (Fig S2 & S6). This highlights that by approaching growth as one trait, and the growth measurements under different stress as realization of the same trait on different environments, in our analysis and then comparing detected QTL/epistatic QTL across environments new hypotheses can be formulated for future, in-depth studies on the shared and unique biological mechanisms involved under various types of stress. In this way, this modeling provides an advanced understanding about the contributions from G by G by E interactions to complex traits variation in general and their influence on the quantitative genetics metrics commonly used to dissect quantitative traits in higher organisms in particular.

*Q3: The difficulty of biological interpretability of the analyses is apparent from the discussion section -- little biological insight emerges about genetic networks in yeast, nor is there concrete new understanding the reader can take away about the genetic features that may shape GxGxE interactions in yeast or other organisms.*

R3: As mentioned in R1 above, our aim with this study was not to provide molecular/functional biological insight, but rather results of value for statistical and quantitative genetics. As stated before, future work to address the molecular basis of the findings would be highly valuable but beyond the current study.

**Reviewer #3:**

*In this manuscript, the authors investigated and evaluated the role of G-by-G-by-E interaction on the complex traits. To explore the role of environment influencing epistatis that can lead to the expression of cryptic variation, the authors reanalyzed a dataset of 4,390 yeast genotyped segregants, which were phenotyped in 20 different conditions. As previously shown, they found that genetic interaction networks are contributing to complex traits. But more interesting, they highlighted and illustrated the importance of dynamics in epistatic network, genetic effects of individual alleles and genetic variance expressed across environments.*

*Overall, the manuscript is very interesting and bring a new insights into the mechanisms involved in the variation of complex traits.*

*I only have a couple of points that need to be addressed:*

Q1: The authors use one cross between two given isolates, namely BY and RM. It is always difficult to know if the obtained results are not specific to the studied cross. This is something that should be discussed in the manuscript.

Q1: We fully agree that a further validation in other crosses, different environments and perhaps even under natural conditions would be very exciting. Hopefully other suitable datasets will be released in the future, allowing us to contribute to this work! This is now discussed in the revised manuscript.

*Q2: As the phenotypes are pre-normalized growth measurements (against growth in a control medium), the authors do not have a direct estimation of G-by-E. Did they try to run the same analysis on the non-normalized dataset? What are the results?*

R2: Unfortunately, the non-normalised data is not publicly available and therefore we were not able to perform this analysis, despite it being of great interest also to us. However, direct estimates for the G-by-E interactions were obtained by fitting a model with strain ID (G) and E as factors, and their interaction, as explanatory variables and the repeated measurements for colony size as the response variable. The resulting ANOVA showed that the sum of squares for E was 0 (due to the normalization), but that the sum of squares from the interaction term was twice as large as that for strain ID. This suggests that the amount of variance explained directly by G was only half of that of G-by-E. The section in the discussion addressing this is highlighted in the revision).

*Q3: In the author summary, the word ‘gene’ is misused – e.g. ‘individual with the same gene might have distinctive phenotypes’.*

R3: Thank you for pointing this out, it has now been changed to ´allele´

*Q4: The discussion is very long and wordy. It could be shortened.*

R4: The revised manuscript has been shortened in several places to make it more focused.

**Reference:**

1.	Rivas E-M, Gil de Prado E, Wrent P, de Silóniz M-I, Barreiro P, Correa EC, et al. A simple mathematical model that describes the growth of the area and the number of total and viable cells in yeast colonies. Lett Appl Microbiol. 2014;59: 594–603. doi:10.1111/lam.12314
