# Supplementary material for: Dynamic genetic architecture of yeast response to environmental perturbation shed light on origin of cryptic genetic variation
Source: PLoS Genet. 2020 May 11;16(5):e1008801. doi: 10.1371/journal.pgen.1008801 (PMC7241848; doi:10.1371/journal.pgen.1008801)
Supplement: Attachment — Submitted filename: Response_to_reviewers_Rev2.docx [file pgen.1008801.s015.docx]

Dear Editor,

First, we would like to thank you and the two reviewers again for providing comments to improve our manuscript. It has now been revised by including results from an additional analysis and a paragraph in the discussion to address the criticism raised by reviewer two.

A detailed response to all the comments, describing how they have been implemented in the revision, is attached. We believe the additional analysis provided in the response to the reviewer, and the discussion on this topic, addresses your concerns. If you have any further questions, comments or concerns, we would be happy to answer these as well.

Best regards,

Yanjun Zan and Örjan Carlborg

**Editorial comments:**

Dear Dr zan,

Thank you very much for submitting your Research Article entitled 'Dynamic genetic architecture of yeast response to environmental perturbation shed light on origin of cryptic genetic variation' to PLOS Genetics. Your manuscript was fully evaluated at the editorial level and by two of the original three independent peer reviewers. The reviewers appreciated the attention to an important topic but identified some aspects of the manuscript that should be improved.

The reviewers are split in their opinion.  Reviewer #2 still thinks that treating growth under all conditions as a single phenotype misses critical underlying complexity and in doing so renders the analysis flawed.  Reviewer #1 is less concerned by this limitation and acknowledges that you aren't trying to explain all of what is happening but rather you're trying to use a single measurable trait to study complex genetics in a way that it not well represented in the literature. It is important to note that both reviewers are exceptionally qualified experts in the field, so I can appreciate both of their perspectives.

R1: We appreciate the thought-provoking criticism raised by the second reviewer regarding the approach to study Genotype by Environment interactions. Our study is at the interface between statistical and experimental quantitative genetics, where there is currently no broadly accepted consensus on how to approach genotype by environment interactions. This type of discussions are therefore very helpful to not only improve the manuscript but also to further our understanding about how scientists with a slightly different background think about and interpret work on the same topic/data/analysis.

Our interpretation of the reviewer comments is that they originate from different views on “what is a trait” that likely originates from different research backgrounds. We arrive at this from quantitative/population genetics backgrounds, where most of our earlier work is on data from animals and plants. Here complex phenotypes, such as flowering time, crop yield, body weight, meat quality, milk production, height, BMI, diabetes, are generally high-level aggregated measurements. Hence, we conceptually accept that they are due to the action and interaction of many unknown and/or unobservable biological processes and an aim of the study is to apply methods that could, at least partially, infer these.

Influenced by our earlier experiences/conceptualizations of complex trait genetics, we here approached colony radius as a way to measure yeast growth embracing that is likely influenced by multiple genes/pathways/networks. As a consequence of this, we expected that there might be differences in which mechanisms that contributed in the different growth environments. By using a forward genetics screen, the major contributing loci to this common growth-measurement across all environments could be identified without the need for assumptions regarding which contributions were common and specific. We next proceeded to additional analyses aiming to reveal where the identified additive/non-additive loci fell on this sliding scale from being common contributors with significant effects in many environments, to having more specific effects in only a few environments. One advantage of the adopted forward genetic approach is thus that it does not require any prior assumptions about which biological mechanisms are expected to contribute to trait variation, or limit explorations to a set of known genes/pathways/networks. Instead, it aims to dissect the genetic basis of quantitative traits and does this from phenotypes obtained using assays that do not specifically target the output of individual biological mechanisms.

An alternative approach to study complex trait genetics, that is more relevant in organisms where more advanced molecular experiments and trait assays are feasible at scale, is the one of reverse genetics. Here, traits/phenotypes can be defined by targeting assays to the specific outputs from introduced/defined polymorphisms affecting individual biological mechanisms. Although this approach has great potential in providing detailed input to the targeted systems, including how the effects of these change across environments, it is more limited in ability to provide the overall view of how the multitude of loci/pathways/networks involved in the regulation of a complex trait combine their effects than the forward genetics screen.

Hence, defining “traits”, by tradition and method, will be different in forward and reverse genetics approaches to study the basis of complex, quantitative traits. While we fully agree with the reviewer that it would be of great interest to study Genotype x Environment interactions in a reverse genetics context with traits defined as outputs from specific biological mechanisms, we believe that the forward genetics approach applied here in parallel to this will lead to the greatest overall gains in knowledge. Unfortunately, proper application of a reverse genetic approach is not possible in this data since it requires a different design and collection of data to start with.

As a proxy for a classic reverse genetics analysis, the reviewer suggests to use statistics in an attempt to partition the variance of the measured complex quantitative phenotype into components intended at representing common and specific genetic contributions to yeast growth in the studied environments. By performing genome scans separately for these components loci contributing to these could be identified. Although we think this is an interesting idea that might add complementary information to the results presented here, the outlined approach and analyses have not been formally developed, applied and tested in this context before. Hence, it would require and deserve proper evaluations beyond the scope of this work to, for example, explore how these statistically inferred components derived from the complex colony size phenotypes reflect the biologically independent mechanisms that they are intended to target. We consider a rapid implementation and application of this approach to this data in response to this review, without the possibility to perform a proper formal evaluation/comparison to a relevant experimental dataset reflecting what it is intended to capture, as something that would distract us more rather than add valuable insights to the current work.

However, inspired by the reviewer suggestion we performed a more classic quantitative genetic correlation analysis of colony sizes measured in the different environments. This as an approach to quantify the shared and unique genetic contributions to growth across them. These analyses showed that, despite the large differences in growth environments, also the genetic correlations across the environments were high and in line with the earlier reported extensive overlaps of both additive loci and active epistatic network interactions (S1-3 Fig) . This was observed not only for environments expected to be similar, but also for growth conditions that are expected to require distinctive physiological responses, suggesting that there is sharing of underlying biological mechanisms also on those cases. These new results are included in the Supplements of the revised manuscript.

In summary, we have now revised the paper to acknowledge the difference in how traits are defined in different fields. Further we also discuss how this impacts the interpretation and expectation of insights gained from the reported results. We have not performed the suggested statistical analysis, as we feel that it needs a thorough formal evaluation before it is published. Instead we performed a more classic genetic correlation analysis, the results from which supported earlier findings of overlaps of loci/interactions across also rather different growth environments, and have included this in the Supplements. We hope that you find these revisions sensible and that the manuscript after this is acceptable for publication

Q2: As a way of threading the needle I think it will be critical to fully acknowledge the limitations described by Reviewer #2 in the text of the article and explain, at least by fully discussing if not by revising the analysis, how the system would be structured if individual growth components were accounted for.

R2: We hope the explanation above in R1, the additional genetic correlation analysis included in the manuscript/discussed in more detail below in the response to reviewer #2, and the revised discussion on this topic in the new manuscript addresses your concerns. If something is still unclear, we are happy to discuss it further with you.

Q3: We therefore ask you to modify the manuscript according to the review recommendations before we can consider your manuscript for acceptance. Your revisions should address the specific points made by each reviewer.

In addition we ask that you:
1) Provide a detailed list of your responses to the review comments and a description of the changes you have made in the manuscript.

R3: Detailed responses to the review comments are included together with a manuscript version where the changes are highlighted.

Q4: 2) Upload a Striking Image with a corresponding caption to accompany your manuscript if one is available (either a new image or an existing one from within your manuscript). If this image is judged to be suitable, it may be featured on our website. Images should ideally be high resolution, eye-catching, single panel square images. For examples, please browse our [archive](http://www.plosgenetics.org/article/browse/volume). If your image is from someone other than yourself, please ensure that the artist has read and agreed to the terms and conditions of the Creative Commons Attribution License. Note: we cannot publish copyrighted images.

R4: Unfortunately, we do not have any such an image to provide.

We hope to receive your revised manuscript within the next 30 days. If you anticipate any delay in its return, we would ask you to let us know the expected resubmission date by email to [plosgenetics@plos.org](mailto:plosgenetics@plos.org).

If present, accompanying reviewer attachments should be included with this email; please notify the journal office if any appear to be missing. They will also be available for download from the link below. You can use this link to log into the system when you are ready to submit a revised version, having first consulted our [Submission Checklist](http://journals.plos.org/plosgenetics/s/submit-now#loc-submission-checklist).

While revising your submission, please upload your figure files to the [Preflight Analysis and Conversion Engine](http://pace.apexcovantage.com/) (PACE) digital diagnostic tool. PACE helps ensure that figures meet PLOS requirements. To use PACE, you must first register as a user. Then, login and navigate to the UPLOAD tab, where you will find detailed instructions on how to use the tool. If you encounter any issues or have any questions when using PACE, please email us at [figures@plos.org](mailto:figures@plos.org).

Please be aware that our [data availability policy](http://journals.plos.org/plosgenetics/s/data-availability) requires that all numerical data underlying graphs or summary statistics are included with the submission, and you will need to provide this upon resubmission if not already present. In addition, we do not permit the inclusion of phrases such as "data not shown" or "unpublished results" in manuscripts. All points should be backed up by data provided with the submission.

PLOS has incorporated [Similarity Check](http://www.crossref.org/crosscheck.html), powered by iThenticate, into its journal-wide submission system in order to screen submitted content for originality before publication. Each PLOS journal undertakes screening on a proportion of submitted articles. You will be contacted if needed following the screening process.

To resubmit, you will need to go to the link below and 'Revise Submission' in the 'Submissions Needing Revision' folder.

Please let us know if you have any questions while making these revisions.

Yours sincerely,

Gregory P. Copenhaver
Editor-in-Chief
PLOS Genetics

**Detailed responses to reviewers**

**Reviewer #1:**

The authors have responded to and clarified my concerns from the previous version.

Q1: I have one suggestion, would it be possible to include the additivity and barcoding/independence analysis as a supplemental section. This analysis will be very helpful for the community when considering population size and locus independence when creating future populations or analyzing existing populations. I personally had not expected the role of environment in structuring the asymptote. This would be very useful for citations and guiding grant proposals.

R1: We have followed this suggestion and included this as a supplemental section to the manuscript.

Response 2:

Q1: As a reviewer I still have major concerns about the appropriateness of the analyses presented in this manuscript. My concerns are both biological and methodological as I'll describe below. I will try not to be "overly-aggressive" in my framing of these concerns, but I feel it necessary to the highlight what I see as a key methodological flaw. I will also note that at the same time I am quite positive about the questions this manuscript sets out to address.

R1: We appreciate the enthusiasm regarding the research question and how the feedback on our approach to study Genotype by Environment interactions has helped us understand the different perspectives on it. Our background is in animal/plant genetics and with this study we are at the interface between statistical and experimental quantitative genetics, where the attempt is to use accepted approaches in the field we come from to new and more powerful data. Your comments made it clear that there is currently no broadly accepted consensus on how to analyze genotype by environment interactions from genotype and phenotype data, but rather that we are all highly influenced by the paradigms of our respective fields. Your criticisms and these further discussions are therefore very helpful to not only improve the manuscript, better frame the descriptions/discussion of the results in the paper to make sense for a broader audience, but also further our own understanding on how the community think about and interpret work on the same topic/data/analysis.

Hence, we fully acknowledge that there are alternative approaches to this problem, and the approach you suggest is likely to provide deeper functional insights to the biological mechanisms underlying genotype x environment interactions. However, given what data is available we believe that the current analysis and results are valid, and that they will be useful for guiding additional future work to approach these mechanisms. Inspired by your comments, we have performed additional analyses to estimate also the genetic correlations between the colony sizes in the different environments and included them in the manuscript. Further, we have revised the discussion to explain and discuss the advantages and shortcomings of our approach in more detail. We hope that this together addresses your concerns.

Q2: My key biological concern can be summarized as "what is a trait?" For an analysis of Genotype by-Environment interactions to make sense, the underlying assumption is that one has measured the same trait under different environmental conditions. Here the trait is ostensibly "growth".

R2: On a conceptual level, we completely agree that “what is a trait” is a central – and at the same time very difficult to standardize – concept in genetics. We approach the definition of “trait” from a forward genetics perspective, i.e. the phenotype first paradigm commonly used in analyses of for example plants, animals and humans. Colony radius was therefore, in the same way as flowering time, crop yield, body weight, meat quality, milk production, human height, BMI, diabetes etc, considered as a high-level aggregated traits measurement. In doing so, it is expected to be the result from the action and interaction of many unknown and/or unobservable biological processes. The aim of the analyses was therefore first to identify loci affecting this common growth-measurement across all environments, and next evaluate whether the identified additive/non-additive loci fall on the sliding scale from being common contributors with significant effects in many environments to be more specific in only a few environments. Hence, rather than attempting to define growth as a trait resulting from (or representing) specific biological/physiological processes, and study the genotype x environment interactions involving these, the forward genetics screen and the subsequent analysis estimates the expected overlap of different processes to a common complex trait measurement across environments.

One advantage of the adopted forward genetic approach is that it does not require any prior assumptions about which biological mechanisms are expected to contribute to trait variation, or limit explorations to a set of known genes/pathways/networks. This makes it possible to dissect the genetic basis of quantitative traits using assays that are not specifically targeting the output of individual biological mechanisms and obtain a general overview of the link between genetic polymorphisms and the composite phenotype. In contrast, the reverse genetic approach will provide more detailed insights to specific biological mechanisms beyond what can be achieved in a forward genetics screen. It is, however, difficult to obtain the same general overview of how loci/pathways/networks combine their effects as in a forward genetics screen. Rather than argue one or the other approach being flawed, we believe that it is a combination of both that will pave the way of future development of the field.

We hope that this response, together with the revisions of the discussion in the manuscript, better explains the context of the current work and why it rather than being considered as flawed could be viewed as an alternative and complementary approach to others more commonly used in studies of yeast.

Q3: However, my concern is that the biological underpinnings of yeast growth in the different treatments is different enough that many of the subsets of "growth" measures should be treated as different traits, absent an analysis that convincingly demonstrates them to share a high degree of similarity (see below for suggestions about one such quantitative model for approaching this). While there may be a "common growth factor" that is shared across the different measures, I argue that for many of the traits the "condition specific factors" may largely outweigh such a common factor.

R3: First, see the conceptual discussion relating to forward and reverse genetics approaches in responses to Q1 and Q2 above.

First, we would like to point out that the forward genetics screen allows us to, instead of assuming/focusing on particular shared biological underpinnings of yeast growth to define a trait, estimate such similarities/differences in the underlying genetics by mapping the genetic architectures in the different environments. This was not clearly highlighted in the earlier manuscript, but is now revised in the updated manuscript. Further, we have also performed additional genetic correlation analyses (described below) to further describe the shared genetics across environments and included these in the revised manuscript.

Previously, we presented the pairwise phenotypic correlations (Figure S2) and number of overlapping additive/non-additive loci (Figure S3) as indicators of the shared genetic architecture of the traits. As shown in Figure S2-S3, the phenotypic correlations ranged from -0.29 to 0.70 and there was a variable degree of overlaps for both additive and non-additive loci, both of which indicates a significant overlap in the genetic architecture of colony size across the measured environment. Inspired by your comments, we now also estimated the pairwise genetic correlations across the all environments. This analysis treats the phenotype measured in the different environments as separate traits and account for the correlation between environments by including a genetic correlation (Falconer, 1952. Introduction to Quantitative Genetics. Harlow: Longmans Green; Eisen et al, 1983, Theoretical and Applied Genetics). This result is summarized in Figure R1 below and included in the revised manuscript as Fig S1. The estimated genetic correlations ranged between -0.47 to 0.88. Together with the overlapping of detected additive/non-additive QTL presented in Figure S2-S3, these results strongly indicate that a shared genetic component not only exist, but also sometimes clearly outweigh the environment specific component. Thus, despite the large differences in growth environments, both high genetic correlations, and considerable overlaps of both additive loci and active epistatic network interactions (S1-3 Fig), were found. This is the case even for growth conditions expected to require distinct physiological responses, suggesting at least some degree of shared sunderlying biological mechanisms. This result has now been included in the revised manuscript and discussion.

*Figure R1. Pairwise Spearman rank correlations among colony sizes measured across the 20 studied media. Numbers in the cells are 100 times the genetic correations. Environments are sorted based on their order after hierarchical clustering based on the phenotypic correlations. The values on the diagnal are 100 x the narrow sense heritibilites.*

Q5: If this is the case, then the fact that the inferred G-by-E interactions differ across treatments is not surprising but is simply a consequence of the fact that the authors are estimating the genetic architecture of what in essence are many different traits.

R5: As described in R4 above, no such assumption was made and actually the results obtained from the analyses of the experimental data do not support this. By treating the observations as the same traits allow us to explore how gradual changes in the environment results in corresponding gradual changes in the underlying genetic architecture defined by the overlaps of both additive effects and non-additive interactions (Figure 3; main manuscript).

Q6: In order to make this argument quantitative, I ask the authors (and Editor) to consider the path analysis/SEM style model below (see embedded image). In this figure, the "Common Growth Factor" and "Shared Growth Factors" represent latent variables that contribute to the variation of each of the individual traits (growth in each of the environments). The unlabeled arrows represent trait specific contributions to each growth trait. An SEM style model like this posits that correlation between any two pairs of traits is a function of the "Common Growth Factor" and any "Shared Growth Factors" that the traits may have in common. Note that models like this are common in the morphometric literature, where the "Common Growth Factor" would be usually replaced by a "General size factor" (e.g. Bookstein, F. L. 1991. Morphometric Tools for Landmark Data. Geometry and Biology. Cambridge University Press: New York).

I suggest that estimating such a model, analyzing the contribution of this "Common Growth Factor" to the variance of each traits, and then comparing the genetic architecture of this "Common Growth Factor" across treatments would be both more biologically and statistically sound approach to analyzing G-by-E interactions. I predict (though can't say for sure not having done the analysis) that an analysis that reflected both the biological complexity of the traits at hand as well as the statistical complexity that describes their covariance structure would lead to greater biological interpretability of the results.

R6: First, we refer to response Q5 describing the additional genetic correlation analyses performed to estimate shared genetics of growth across environments.

Next, we appreciate this detailed illustration of the SEM model and will with great interest follow the development of it in the context of genotype x environment analyses, and its implementation in forward genetic screens for this purpose. However, although the implementation of this statistical approach appears rather straight forward, it deserves a proper evaluation both theoretically and by application to real data beyond what can be done in this manuscript. Since they aim to disentangle shared and unique biological contributions to the complex trait, it would be highly beneficial to combine them with further experiments where the basis of the studied trait was actually known. Without this, it is difficult to predict how these statistically inferred components of the phenotypes related to the underlying biology and whether they are a good proxy for the reverse genetics analysis.

We hope the explanations above, additional analysis included in the manuscript, and the revisions of the discussion section in the manuscript has addressed your concerns to the point where the manuscript is now acceptable for publication.
